# Clinical Characteristics in Patients with Triple Negative Breast Cancer

**DOI:** 10.1155/2017/1796145

**Published:** 2017-08-17

**Authors:** Janet Yeh, Jennifer Chun, Shira Schwartz, Annie Wang, Elizabeth Kern, Amber A. Guth, Deborah Axelrod, Richard Shapiro, Freya Schnabel

**Affiliations:** ^1^Department of Surgery, New York University Langone Medical Center, New York, NY, USA; ^2^School of Medicine, New York University Langone Medical Center, New York, NY, USA; ^3^Drexel University, School of Medicine, Philadelphia, PA, USA

## Abstract

**Purpose:**

The purpose of this study was to compare and contrast the clinical characteristics of the triple negative breast cancer (TNBC) and non-TNBC patients, with a particular focus on genetic susceptibility and risk factors prior to diagnosis.

**Methods:**

Our institutional database was queried for all patients diagnosed with invasive breast cancer between January 2010 and May 2016.

**Results:**

Out of a total of 1964 patients, 190 (10%) patients had TNBC. The median age for both TNBC and non-TNBC was 59 years. There was a significantly higher proportion of African American and Asian patients with TNBC (*p* = 0.0003) compared to patients with non-TNBC. BRCA1 and BRCA2 were significantly associated with TNBC (*p* < 0.0001, *p* = 0.0007). A prior history of breast cancer was significantly associated with TNBC (*p* = 0.0003). There was no relationship observed between TNBC and a history of chemoprevention or patients who had a history of AH or LCIS.

**Conclusions:**

We found that having Asian ancestry, a prior history of breast cancer, and a BRCA1 or BRCA2 mutation all appear to be positively associated with TNBC. In order to develop more effective treatments, better surveillance, and improved prevention strategies, it is necessary to improve our understanding of the population at risk for TNBC.

## 1. Introduction

Triple negative breast cancer (TNBC) is the subtype of breast cancer that does not overexpress human epidermal growth factor 2 receptors (HER2), while also lacking expression of estrogen receptors (ER) and progesterone receptors (PR). TNBC, which accounts for an estimated 15–20% of invasive breast cancers [1–3], has been associated with rapid growth, distant metastasis, and shorter overall and relapse-free survival when compared to other breast cancer subtypes across multiple studies [4–6].

Much of the literature surrounding TNBC has been focused on identifying populations at risk. In particular, BRCA1 and BRCA2 genotypes have been shown to predispose carriers to TNBC [5, 7–11]. The BRCA genes encode tumor suppressors that repair DNA damage by homologous recombination; when mutated, the carrier is susceptible to breast and ovarian cancer [12]. Studies suggest 10.6–30.9% of TNBC patients are carriers for deleterious BRCA1 and BRCA2 germline mutations, especially BRCA1 [7, 10, 13, 14]. Routine BRCA mutation testing is not recommended for all patients with breast cancer due to its high cost and the low prevalence of mutations [15–17]. However, the National Comprehensive Cancer Network (NCCN) guidelines recommend referral for consideration of genetic counseling for women 60 years of age or younger with TNBC [13, 18]. Recent reports have also shown strong associations between TNBC and diagnosis of breast cancer at a younger age [19, 20].

Another population with increased incidence of TNBC is African American patients [1, 21–23]. Carey et al. devised a case-control study in North Carolina in which African American patients were overrepresented to allow for statistical comparison to mostly Caucasian patients; this landmark study found that 39% of African American patients presented with TNBC, compared to 16% of non-African American patients [1]. Amirikia and colleagues analyzed the prevalence of TNBC among non-Hispanic White, non-Hispanic Black, and Hispanic patients in the California Cancer Registry; they confirmed the association and asserted a steeper rise in incidence of TNBC with age among African American women [21]. Multiple locoregional studies demonstrate that TNBC comprises 20–40% studies of African American breast cancer patients [1, 21–24]. However, there have not been any American studies analyzing TNBC in the Asian population. Among 972 breast cancer patients at the Dr. B. Borooah Cancer Institute in India, 31.9% were defined as TNBC [25]. Additionally, the University Malaya Medical Center in Malaysia reports a 17.6% incidence of TNBC among 1147 breast cancer patients of Chinese, Malay, and Indian descent [26]. From these international reports, we suspect that TNBC may affect a large proportion of Asian American patients.

The present study was designed to compare and contrast the clinical characteristics of the TNBC and non-TNBC patients in the New York University Breast Cancer Database (BCD), with a particular focus on genetic susceptibility and risk factors prior to cancer diagnosis.

## 2. Methods

### 2.1. Study Participants

The Breast Cancer Database (BCD) is a longitudinal study that was established at our institution in January 2010. All patients undergoing definitive breast cancer surgery for a newly diagnosed breast cancer at our institution are eligible to enroll in the BCD. The variables collected for the database include information on personal and family history, screening history, methods of diagnosis, tumor histology and stage at diagnosis, details of treatment, and outcomes. All clinical data are obtained from detailed questionnaires that are filled out at the time of surgery and from a review of electronic medical records. The Breast Cancer Database was queried for all patients who were diagnosed with invasive breast cancer between January 2010 and May 2016. This study was approved by the Institutional Review Board.

### 2.2. Statistical Analyses

Descriptive statistics were used to summarize the data and to see the distribution of the variables between patients with TNBC and patients with non-TNBC. The variables of interest included age, race, personal and family history of breast cancer, BRCA1 and BRCA2 status, and tumor characteristics. Pearson's chi-square and Fisher's exact tests were used to assess any associations between the categorical variables of interest and TNBC status with a significance level of 0.05. Logistic regression was used to test for any associations between the continuous predictor variables, age and tumor size, and TNBC. All analyses were performed using SAS version 9.3 (SAS Institute Inc., Cary, NC).

## 3. Results

During the study period, a total of 1964 patients with invasive breast cancer were enrolled in the Breast Cancer Database. The majority of patients in the study cohort were Caucasian (76%). The median age was 59 years (22–95 years). There were 31 (5%) patients who were BRCA1 positive and 29 (5%) who were BRCA2 positive. As expected, the majority of breast cancer patients had tumors that were early stage, stage I or II, (91%) and of ductal histology (81%).

Out of a total of 1964 patients, 190 (10%) patients had TNBC and 1774 (90%) had non-TNBC. The median age for both TNBC and non-TNBC was 59 years. There was a significantly higher proportion of African American and Asian patients with TNBC (*p* = 0.0003) compared to patients with non-TNBC (Table 1). BRCA1 and BRCA2 were significantly associated with TNBC (*p* < 0.0001, *p* = 0.0007). A prior history of breast cancer was significantly associated with triple negative histology in our study (*p* = 0.0003). There was no relationship observed between TNBC status and a history of chemoprevention. Likewise, there was no relationship between TNBC status and patients who had a history of AH or LCIS (Table 1). When compared to patients with non-TNBC, patients with TNBC were more likely to have undergone neoadjuvant treatment (*p* < 0.0001). As compared to patients with non-TNBC, patients with TNBC were also more likely to have later stage disease (*p* = 0.0001), invasive ductal carcinomas (*p* < 0.0001), and higher Ki-67 (*p* < 0.0001) (Table 2).

## 4. Discussion

The results of our study both confirm known associations of TNBC and identify significant notable findings specific to our TNBC population. Similar to findings in previous studies, our TNBC patients had an increased proportion of African American patients and a higher percentage of BRCA mutation carriers when compared to the non-TNBC population. A significant association between TNBC and previous history of breast cancer was also seen. No association was found between TNBC and prior history of atypical hyperplasia or LCIS. Contrary to many previously published studies, we found a higher proportion of Asians with TNBC. We also found that the median age in both TNBC and non-TNBC cohorts was 59 years.

TNBC has been shown to be associated with a 10.6–30.9% carrier rate of deleterious BRCA1 and BRCA2 mutations [7, 10, 13, 14]. Our data is consistent with an increased number of carriers in our TNBC population. This may be due to the unique racial distribution of our patients. Internationally, there are differences in BRCA1 and 2 prevalence; for example, among 190 TNBC patients in Mexico City, 23% were found to have a founder BRCA1 mutation (BRCA1 ex9-12del) [27], while BRCA risk calculators developed in Caucasian populations consistently underestimate BRCA risk in Asian patients [28]. We postulate that the higher proportion of Asian American patients in our database may account for this discrepancy with previous literature. The NCCN guidelines recommend that all women with TNBC and 60 years of age or younger should undergo genetic counseling [18]; moreover, our study supports the NCCN guideline and underlines the importance of genetic counseling for TNBC patients.

There was a significant association between TNBC and individuals of African American and Asian heritage within our cohort. We identified 18% and 13% of individuals in the TNBC population as African American and Asian, respectively. African American and Asian patients each comprise 8% of the non-TNBC population. The association between African American patients and TNBC is well established in several studies [1, 21–23]. However, there is insufficient data on the association of TNBC with individuals of Asian descent in current medical literature. There may be distinct Asian populations that are more susceptible to TNBC than others, which would explain our findings. Jack et al. showed a similar association between TNBC and a South Asian population [29], while Lakshmaiah et al. showed a higher incidence of TNBC in an Indian population compared to Western populations [30]. Thus, further assessments should be performed to differentiate Asian populations with increased associations with TNBC.

The average age of diagnosis for TNBC has been shown to be 5–10 years younger than patients with non-TNBC [11]. Of note, the median age was the same in our TNBC and non-TNBC cohorts. The older median age of our TNBC patients may be related to the increase of patients with a prior personal history of breast cancer and may be attributed to the prior use of endocrine therapy. It has been shown that endocrine therapy reduces the risk of ER positive breast cancer [31–33]. On long-term follow-up, the likelihood of TNBC is shown to increase. However, we were not able to demonstrate a relationship between having TNBC and prior use of endocrine therapy in our cohort. This may be due to low numbers of patients with a prior history of chemoprevention.

AH and LCIS are known global risk factors linked to the development of breast cancer in women [34–36]. Our study confirmed a lack of association between TNBC and a prior history of AH or LCIS. This may be because most women with AH and LCIS decline chemoprevention and a large proportion of patients who start chemoprevention do not complete the course due to side effects [37, 38]. Therefore, patients with a prior history of AH and LCIS have lower exposure to endocrine therapies than those with prior history of breast cancer. Additionally, it has been theorized that there may be a different pathogenesis of TNBC compared to other subtypes of breast cancer [39].

## 5. Conclusions

While our study confirmed known characteristics associated with TNBC, it also highlighted unexpected results that merit further investigation. Based on our TNBC population, we found that having Asian ancestry, a previous personal history of breast cancer, or a germline BRCA mutation all appear to be positively associated with TNBC. This finding further supports the revised NCCN guidelines that recommend women 60 years of age or younger with TNBC to be referred for consideration of genetic counseling. In addition, there was a lack of association between TNBC and personal history of AH and LCIS.

In order to develop more effective treatments, better surveillance, and improved prevention strategies, it is critical to improve our understanding of the risk factors that are associated with the development of triple negative breast cancer.

## Figures and Tables

**Table 1:** Clinical characteristics.

Variables	TNBC (*N* = 190, 10%)	%	Non-TNBC (*N* = 1774, 90%)	%	*p* value
*Median age, in years*	59 (25–92)		59 (22–95)		0.52
*Race*					
African American	31	16	147	8	0.0003
Asian	23	12	152	9	
White	122	64	1363	77	
Hispanic	11	6	103	6	
Other	3	2	9	1	
*AH*					
No	188	99	1741	98	0.57
Yes	2	1	33	2	
*LCIS*					
No	190	100	1751	99	0.16
Yes	0	0	23	1	
*Previous history of breast cancer*					
No	156	82	1605	90	0.0003
Yes	34	18	169	10	
*Family history of breast cancer*					
No	144	76	1352	76	0.90
Yes	46	24	442	24	
*BRCA1*					
Negative	65	82	481	97	<0.0001
Positive	14	18	17	3	
Unknown/not tested	111	—	1276	—	
*BRCA2*					
Negative	68	86	480	96	0.0007
Positive	11	14	18	4	
Unknown/not tested	111	—	1276	—	
*Chemoprevention*					
No	179	94	1695	96	0.40
Yes	11	6	79	4	

**Table 2:** Tumor characteristics.

Variables	TNBC (*N* = 190, 10%)	%	Non-TNBC (*N* = 1774, 90%)	%	*p* value
*Neoadjuvant*					
No	157	83	1669	94	<0.0001
Yes	33	17	105	6	
*Median tumor size (cm)*	1.6 (0.1–9.4)		1.3 (0.04–12.5)		0.06
*Stage*					
I	101	53	1111	63	0.0001
IIA, IIB	55	29	513	29	
IIIA, IIIB, IIIC	33	17	138	8	
IV	1	1	12	1	
*Histology *					
IDC	178	94	1422	80	<0.0001
ILC	5	3	242	14	
Invasive other	7	4	110	6	
*Median Ki-67*	60 (1–99)		10 (1–99)		<0.0001
